# Formaldehyde and Glutaraldehyde Inactivation of Bacterial Tier 1 Select Agents in Tissues

**DOI:** 10.3201/eid2505.180928

**Published:** 2019-05

**Authors:** Jennifer Chua, Joel A. Bozue, Christopher P. Klimko, Jennifer L. Shoe, Sara I. Ruiz, Christopher L. Jensen, Steven A. Tobery, Jared M. Crumpler, Donald J. Chabot, Avery V. Quirk, Melissa Hunter, David E. Harbourt, Arthur M. Friedlander, Christopher K. Cote

**Affiliations:** United States Army Medical Research Institute of Infectious Diseases, Frederick, Maryland, USA

**Keywords:** Bacillus anthracis, Burkholderia pseudomallei, Burkholderia mallei, Francisella tularensis, Yersinia pestis, Tier 1, Select Agents, guinea pigs, rabbits, formalin, paraformaldehyde, formaldehyde, glutaraldehyde, sterility, inactivation, viability testing, neutralization methods, lung, liver, spleen, skin, outer ear pinna, spores, bacteria

## Abstract

For safety, designated Select Agents in tissues must be inactivated and viability tested before the tissue undergoes further processing and analysis. In response to the shipping of samples of “inactivated” *Bacillus anthracis* that inadvertently contained live spores to nonregulated entities and partners worldwide, the Federal Register now mandates in-house validation of inactivation procedures and standardization of viability testing to detect live organisms in samples containing Select Agents that have undergone an inactivation process. We tested and validated formaldehyde and glutaraldehyde inactivation procedures for animal tissues infected with virulent *B. anthracis*, *Burkholderia pseudomallei*, *Francisella tularensis*, and *Yersinia pestis*. We confirmed that our fixation procedures for tissues containing these Tier 1 Select Agents resulted in complete inactivation and that our validated viability testing methods do not interfere with detection of live organisms. Institutions may use this work as a guide to develop and conduct their own testing to comply with the policy.

Despite being a disease of antiquity, anthrax remains a public health concern and is considered a reemerging threat in developed countries, in part because of bioterrorism (*1*). Threats of bioterrorism and the ease of global travel have led nations to also be concerned about diseases such as tularemia, plague, and melioidosis (*1*). In addition, natural outbreaks and the global distribution and endemic nature of these bacteria continue to be subjects of public health and biodefense research.

In the United States, biological agents and toxins with the potential to pose a severe threat to public health and safety are overseen by the Federal Select Agents Program (https://www.selectagents.gov), a joint program of the Centers for Disease Control and Prevention/Division of Select Agents and Toxins and the US Department of Agriculture Animal and Plant Health Inspection Service/Agriculture Select Agent Services. Agents that pose a particularly high risk to humans are classified as Tier 1 Select Agents; *Bacillus anthracis* is a Tier 1 Select Agent.

Work with Select Agents necessitates complete inactivation because these organisms can cause serious illness or death and could potentially endanger public health through accidental infection of laboratory workers. In 2015, virulent *B. anthracis* samples thought to be completely inactivated by irradiation were unwittingly sent to unregistered laboratories (*2*). These samples, containing spores produced to support research and development for detection and medical countermeasures, were sent to domestic and international entities not under the purview of the Federal Select Agent Program. Although the samples contained low numbers of live organisms and did not pose a serious risk, this event was a breach in a regulation intended to restrict access to the pathogen and safeguard public health. Investigations at the transgressing facility revealed that *B. anthracis* could be cultured from multiple γ-irradiated batches of spores (*2*). Another troubling aspect was the failure of viability tests, performed after irradiation, to detect viable organisms. Thus, the US Department of Defense developed a well-characterized and reproducible method for inactivating *B. anthracis* spores with irradiation (*3*). In addition, all validation of inactivation procedures and viability testing involving Tier 1 Select Agents at each institution is now mandated by federal regulation (*4*).

A common way to inactivate infectious agents in tissues before histologic analysis is formaldehyde fixation, coincidentally first characterized in 1893 with the fixation of *B. anthracis–*infected tissue (*5*). Glutaraldehyde, the use of which was described decades later in 1963 (*6*), is commonly used to inactivate samples for electron microscopy (EM) analysis. These related aldehydes cross-link primary amines and other reactive groups in proteins, fatty acids, and nucleic acids, thereby halting biochemical reactions and placing cellular structures in permanent stasis resembling structures found in living tissue (*5*,*7*–*9*). Formaldehyde molecules are small and diffuse quickly but fix tissue slowly (*7*,*10*). An attractive property of formaldehyde fixation is that it is partially reversible and some denatured antigens can be retrieved to be again recognized by antibodies (*11*). In contrast, the larger glutaraldehyde molecules fix tissues quickly and irreversibly but do not penetrate thick tissues well.

Despite a century of use, formaldehyde inactivation of tissues containing Select Agents has been described in few reports (*12*,*13*). Frequently, studies test only a small fraction of tissue for complete inactivation (*14*), which carries a risk of concealing low numbers of viable organisms in the remaining sample. The process of fixation, and concurrently that of inactivation, is dependent on variables such as time, pH, temperature, fixative concentration, and tissue size/composition (*10*,*15*–*17*). The desire to accelerate the fixation process for faster workflow (*15*,*17*) increases the risk for incomplete inactivation. Because *B. anthracis* spores in particular are hardy (i.e., resistant to heat, radiation, and chemicals) (*1*,*18*), their inactivation is more difficult.

The detection of viable organisms in partially inactivated tissues relies on the organisms’ ability to proliferate when placed in rich growth media. However, fixative is probably retained internally in tissues, potentially interfering with growth during viability testing. The key function of a viability test is to detect viable organisms by encouraging the proliferation of any organisms, live or injured, that may have survived the inactivation procedure. Testing for viability requires the removal or neutralization of the inactivating agent that may interfere with growth. Removing or neutralizing chemical fixatives is particularly important because their presence might restrict organism proliferation at low, nonlethal concentrations. Similar to the time required for penetration into thick tissues (*10*), adequate time for washing must be provided to allow for outward diffusion. As an alternative, fixative in tissue, including any inhibitory components from the tissue itself, can be diluted in a large volume of medium used for the viability test. Tissue disaggregation or homogenization also exposes potentially live organisms deep within tissues to nutrient-rich medium, allowing growth.

We tested and validated inactivation procedures that used formaldehyde with or without glutaraldehyde on lung, liver, spleen, and skin from infected animals destined for microscopic analyses. Our validation procedure is aligned with requirements set forth by the Centers for Disease Control and Prevention (CDC), under the US Health and Human Services, as mandated in the Federal Register (42 CFR §73) (*4*). We confirmed that our inactivation procedures for tissue fixation resulted in complete inactivation and validated neutralization procedures for viability testing. This work specifically focused on the agents that cause anthrax, melioidosis, tularemia, and plague but could be applicable to others. Because we demonstrated fixation of highly resistant spores, this work could also be applied to unknown or undetermined etiologic agents with uncharacterized properties that cause other emerging infectious diseases.

## Materials and Methods

### Bacterial Strains and Culture

We placed spores of *B. anthracis* Ames (pXO1+/pXO2+), Sterne, and ANR (pXO1+/pXO2–) in Leighton and Doi broth (*19*) or on NBY agar plates (*20*) and purified them with Omnipaque (GE Healthcare, https://www.gehealthcare.com) as previously described (*21*). All spores were heated for 30 min at 65°C before animal infection. Bacilli were grown in brain heart infusion broth (Difco; Becton, Dickinson and Company, http://www.bd.com), on tryptic soy agar, or on sheep blood agar (SBA) plates (Remel ThermoFisher Scientific, https://www.thermofisher.com) at 37°C. *Burkholderia pseudomallei* 1026b and *B. pseudomallei* 82 (purine auxotroph) were grown in Luria Bertani (Lennox) broth (Difco) with 4% glycerol (Sigma Aldrich, https://www.sigmaaldrich.com) (*22*) or on SBA plates. *Francisella tularensis* Schu S4 and live vaccine strain were grown in either brain heart infusion broth with 1% IsoVitaleX (Becton, Dickinson and Company) or on chocolate agar plates (Remel) at 37°C. *Yersina pestis* CO92 and *Y. pestis* Pgm–/pPst– were grown in heart infusion broth with 0.2% xylose (Sigma Aldrich) or on SBA plates at 28°C. All bacterial strains were from the collection at the US Army Medical Research Institute of Infectious Diseases (Frederick, MD, USA).

### Chemical Fixatives

The standard fixative for tissue destined for light microscopy analyses is 4% formaldehyde, which is used interchangeably with 10% neutral buffered formalin, 10% buffered formalin phosphate (Fisher Chemical, https://www.fishersci.com), or 4% paraformaldehyde. Immediately before its use, we used phosphate-buffered saline (PBS), pH 7.4, to dilute 16% paraformaldehyde (Electron Microscopy Sciences, https://www.emsdiasum.com) to 4% paraformaldehyde. We fixed tissues destined for EM with 4% paraformaldehyde and 1% glutaraldehyde (Electron Microscopy Sciences) in 0.1 M sodium cacodylate (Sigma Aldrich) buffer (*14*). This combination is referred to as EM fixative.

### Fixative Removal from Tissue and Formaldehyde Sensitivity Assays

Because of their size and the amount of tissue that can be obtained, we used guinea pigs for these assays. We harvested spleen, liver, outer ear pinna, and skin from euthanized guinea pigs. To enable effective penetration of fixative, we excised tissues <10 mm in 1 dimension. Samples were incubated in fixative (>1:10 wt/vol) at ambient temperature for various times. To allow for outward diffusion of fixative, we soaked samples in PBS/water; ear and spleen required longer submersion to support bacterial growth. Tissues were cut into smaller pieces, ground with a homogenizer (Pro200; Pro Scientific, https://proscientific.com), and then transferred to broth (>1:50 wt/vol). In accordance with CDC policy on the neutralization method, we split the broth into 2 aliquots: 1 was inoculated with 5 × 10^3^ Sterne spores and the other was left as is. The 7-day broth-to-plate viability test for *B. anthracis* was performed by culturing in broth (>1:10 wt/vol) followed by solid medium (>100 µL), each incubated for 7 days at 37°C (*23*). To detect growth in broth, we read optical densities at 620 nm by using a spectrophotometer (Genesys 20, ThermoFisher Scientific).

To test genus-specific sensitivity to formaldehyde, we used *B. pseudomallei* 1026b, *B. anthracis* Sterne, *B. pseudomallei* 82, *F. tularensis* live vaccine strain, and *Y. pestis* Pgm–/pPst–. Strains were incubated in broth with formaldehyde (10% neutral buffered formalin diluted 10-fold from 1:10 to 1:10,000) and shaken for up to 5 days. The starting inoculum was plated for CFU.

### Animal Challenges

To limit the number of animals used, we repurposed Hartley guinea pig survivors **(**Table 1); repurposing was deemed appropriate because of the short duration of the study and because dissemination of bacteria into organs was not required. After administering an intramuscular injection of ketamine, acepromazine (both from Vedco, https://www.vedco.com), and xylazine (Akorn, Inc., http://www.akorn.com), we injected the guinea pigs intradermally at several demarcated locations with 2 × 10^8^ *B. anthracis* Ames spores. To maximize the number of ungerminated spores, we collected whole skin samples at 2 hours after challenge.

**Table 1:** Formaldehyde fixation of *Bacillus anthracis* Ames–infected skin tissue from guinea pigs in study of inactivation of bacterial Tier 1 Select Agents*

Fixation time, d, no. samples	Maximum tissue weight, g	Maximum tissue size, mm	Total CFU recovered/g	Sample inactivation†	Positive control
7, 3	2.04	20 × 30 × 3	6.15 × 10^7^	+/+‡	+/+
14, 3	1.94	23 × 28 × 3	5.41 × 10^7^	–/–	+/+
21, 3	2.55	28 × 28 × 3	4.86 × 10^7^	–/–	+/+

*Infected skin tissues from 3 guinea pigs were fixed for 7, 14, or 21 d in 4% paraformaldehyde, rinsed for 30 min in phosphate-buffered saline, and then homogenized. The maximum tissue weight, size, and CFU recovered per group are listed. +/– indicates presence or absence of growth in broth/on plates. †100% of sample tested. ‡Of 3 samples, 1 was positive for growth.

For spleen, liver, and lung tissue collection, we used <4 naive Strain 13 guinea pigs per Select Agent (Tables 2,3). To maximize spores in the lungs, we administered *B. anthracis* Ames spores to the guinea pigs by the inhalation route (*24*); to enable rapid dissemination to the spleen and liver, we also administered them by the intramuscular route (*25*). In a separate iteration, rabbits were exposed to aerosolized *B. anthracis* Ames spores and lung samples were obtained through tissue sharing.

**Table 2:** Formaldehyde fixation of infected spleen, liver, and lung tissue from guinea pigs in study of inactivation of bacterial Tier I Select Agents*

Infectious agent, tissue type	Maximum tissue weight, g	Maximum tissue size, mm	Maximum CFU/g	Fixation time, d	Sample inactivation	Positive control
*Bacillus anthracis* Ames (inhalational and intramuscular), n = 3						
Spleen	1.04	20 × 20 × 6	1.1 × 10^9^	13	–/–†	+
Liver	1.78	20 × 10 × 10	9.8 × 10^7^	13	–/–†	+
Lung	1.14	20 × 20 × 10	2.1 × 10^8^; 5.5 × 10^4^ (heat resistant)	13	–/–†	+
*Burkholderia pseudomallei* 1026b, n = 4						
Spleen	0.81	20 × 15 × 10	7.5 × 10^8^	13	–/–‡	+
Liver	2.15	20 × 15 × 10	1.4 × 10^7^	13	–/–‡	+
Lung	1.56	20 × 15 × 10	4.0 × 10^7^	17–18	–/–‡	+
*Francisella tularensis* Schu S4, n = 3						
Spleen	1.50	15 × 15 × 10	9.3 × 10^9^	13	–/–§	+
Liver	2.29	25 × 13 × 11	9.5 × 10^7^	13	–/–¶	+
Lung	1.30	15 × 13 × 11	4.3 × 10^8^	13	–/–¶	+
*Yersina pestis* CO92, n = 3						
Spleen	0.96	15 × 10 × 8	1.4 × 10^10^	13	–/–§	+
Liver	2.09	30 × 15 × 10	1.1 × 10^9^	13	–/–¶	+
Lung	1.81	25 × 20 × 15	7.0 × 10^8^	13	–/–¶	+

*<4 guinea pigs were infected with 1 Select Agent. Tissues were excised and then measured, weighed, and plated for CFU. Tissues were fixed in 10% neutral buffered formalin for the indicated time, rinsed for 30 min in phosphate-buffered saline, and then homogenized. +/– indicates presence or absence of growth in broth/on plates. †100% of sample tested. ‡50% of sample tested. §90% of 1 sample tested, 10% of others tested. ¶10% of sample tested.

**Table 3:** Electron microscopy fixation of infected spleen, liver, and lung tissue from guinea pigs in study of inactivation of bacterial Tier 1 Select Agents*

Infectious agent, tissue type	Maximum tissue weight, g	Maximum tissue size, mm	Maximum CFU/g	Fixation time, d	Sample inactivation	Positive control
*Bacillus anthracis* Ames (inhalational and intramuscular), n = 3						
Spleen	0.15	10 × 3 × 2	1.1 × 10^9^	6	–/–†	+
Liver	0.18	9 × 5 × 2	9.8 × 10^7^	6	–/–†	+
Lung	0.08	10 × 4×x 2	2.1 × 10^8^; 5.5 × 10^4^ (heated)	6	–/–†	+
*Burkholderia pseudomallei* 1026b, n = 4						
Spleen	0.09	8 × 4 × 2	7.5 × 10^8^	7	–/–‡	+
Liver	0.16	10 × 6 × 2	1.4 × 10^7^	7	–/–‡	+
Lung	0.09	10 × 4 × 2	4.0 × 10^7^	7	–/–‡	+
*Francisella tularensis* Schu S4, n = 3						
Spleen	0.15	6 × 6 × 1	9.3 × 10^9^	6	–/–§	+
Liver	0.15	11 × 5 × 1	9.5 × 10^7^	6	–/–¶	+
Lung	0.08	7 × 6 × 1	4.3 × 10^8^	6	–/–¶	+
*Yersina pestis* CO92, n = 3						
Spleen	0.11	7 × 5 × 1	1.4 × 10^10^	6 or 8	–/–§	+
Liver	0.07	7 × 4 × 1	1.1 × 10^9^	6 or 8	–/–¶	+
Lung	0.07	7 × 6 × 1	7.0 × 10^8^	6 or 8	–/–¶	+

*<4 guinea pigs were infected with 1 Select Agent. Tissues were excised and then measured, weighed, and plated for CFU. Tissues were fixed in EM fixative for the indicated time, rinsed for 30 min in phosphate-buffered saline, and then homogenized. +/– indicates presence or absence of growth in broth/on plates. †100% of sample tested. ‡50% of sample tested. §90% of 1 sample tested, 10% of others tested. ¶10% of sample tested.

*B. pseudomallei* 1026b (*26*), *F. tularensis* Schu S4 (*27*), and *Y. pestis* CO92 (*28*) were grown until mid-log phase. Bacterial doses and the infection routes used were based on previous studies (Table 4) (*26*,*29*–*31*). To minimize animal pain or distress, we administered meloxicam/buprenorphine (*32*). Animals were observed at least twice daily, and when they were moribund, they were euthanized with pentobarbital (Vortech Pharmaceuticals, Ltd., http://www.vortechpharm.com).

**Table 4:** Routes of infection and delivered doses of Select Agents used to infect guinea pigs and rabbits for tissue collection in study of inactivation of bacterial Tier 1 Select Agents

Bacteria	Route of infection	Delivered dose, CFU	Animal model	Time of collection	Tissue collected
*Bacillus anthracis* Ames (spores)	Intradermal	2.7 × 10^8^	Guinea pigs-Hartley	2 h after challenge	Skin
	Inhalational and/or intramuscular, respiratory	4.9 × 10^6^ and 1.0 × 10^3^ respiratory	Guinea pigs-Strain 13	Late-stage disease	Lung, liver, spleen
	Inhalational	1.0 × 10^7^	Rabbits-New Zealand White	24 h after challenge	Lung
*Burkholderia pseudomallei* 1026b	Intraperitoneal	9.0 × 10^2^	Guinea pigs-Strain 13	Late-stage disease	Lung, liver, spleen
*Francisella tularensis* Schu S4	Subcutaneous	8.6 × 10^2^	Guinea pigs-Strain 13	Late-stage disease	Lung, liver spleen
*Yersina pestis* CO92	Subcutaneous	7.0 × 10^4^	Guinea pigs-Strain 13	Late-stage disease	Lung, liver, spleen

In compliance with the Animal Welfare Act, Public Health Service policy, and other federal statutes and regulations pertaining to animals and experiments involving animals, we conducted our research under an Institutional Animal Care and Use Committee–approved protocol. The facility where this research was conducted is accredited by the Association for Assessment and Accreditation of Laboratory Animal Care, International and adheres to principles stated in the Guide for the Care and Use of Laboratory Animals, National Research Council, 2011 (https://www.aaalac.org; https://grants.nih.gov/grants/olaw/guide-for-the-care-and-use-of-laboratory-animals.pdf).

### Inactivation Procedures and Viability Testing

Skin injection sites from guinea pigs infected with Ames spores were incubated in fixative (1:10 wt/vol) at ambient temperature. Lungs, livers, and spleens from euthanized or recently dead guinea pigs and rabbits were excised, divided into several pieces per tissue type, and submerged in fixative for various times. To remove excess fixative, we soaked fixed tissues for 30 min and homogenized them. The entire homogenate volume was subjected to 7-day broth-to-plate viability testing, as previously described. To provide a positive control, we inoculated an additional sample with spores.

For *B. pseudomallei* 1026b, half of the homogenate was subjected to viability testing and the other half was reinoculated with live organism to serve as a positive control. Most tissue homogenates of *Francisella* and *Yersinia* were tested by using 10% of samples because of the necessary dilution in 10 L of broth for growth; however, spleen tissues with the highest bacterial load were further tested by using 90% of the samples in that volume (10% was reinoculated for positive control). *Burkholderia*, *Francisella*, and *Yersinia* homogenates were incubated in broth and then incubated on solid medium at the appropriate temperature for 3–4 days each.

## Results

Spores were able to germinate and grow in the presence of fixed skin or liver when the tissue was washed for a short time (45 min) (Table 5). In contrast, growth did not occur in ears or spleens fixed for a longer time (14–21 d) but washed for a short time. When washing was extended for a longer time (24 h), spleen tissue again permitted growth (Tables 5, 6), suggesting that the fixative was able to adequately diffuse out of the tissue with longer washing.

**Table 5:** Effect of residual formaldehyde in tissue from guinea pigs on the growth of *Bacillus anthracis* Sterne in study of inactivation of bacterial Tier I Select Agents*

Tissue type, no. samples	Fixation time, d	Incubation time for tissue in PBS/water	Sample inactivation†	Positive control
Skin, 2	2	45 min	–/–	+/+
Skin, 2	14	45 min	–/–	+/+
Skin, 2	21	45 min	–/–	+/+
Ears, 2	2	45 min	–/–	+/+
Ears, 2	14	45 min	–/–	–/–
Ears, 2	21	45 min	–/–	–/–
Spleen, 3	2	45 min	–/–	+/+
Spleen, 3	17	45 min	–/–	–/–
Spleen, 3	17	24 h	–/–	+/+
Liver, 3	2	45min	–/–	+/+
Liver, 3	17	45 min	–/–	+/+‡
Liver, 3	17	24 h	–/–	+/+

*Different fixation times (2, 14, 17, or 21 d) and wash times (45 min or 24 h) for skin, outer ear pinna, spleen, and liver result in the different ability of Sterne spores to germinate and grow in broth used for viability testing. +/− indicates presence and/or absence of growth in broth/on plates; PBS, phosphate-buffered saline. †50% of tissue tested. ‡Of 3 samples, 2 were positive for growth and 1 was inhibited by residual formaldehyde.

**Table 6:** Effect of residual electron microscopy fixative in tissue from guinea pigs on the growth of *Bacillus anthracis* Sterne in study of inactivation of bacterial Tier I Select Agents*

Tissue type, no. samples	Fixation time, d	Incubation time for tissue in PBS/water, h	Sample inactivation†	Positive control
Spleen, 3	14	24	–/–	+/+
Spleen, 3	21	24	–/–	+/+
Liver, 3	14	24	–/–	+/+
Liver, 3	21	24	–/–	+/+

*Spleen and liver tissues fixed for 14 or 21 d and washed for 24 h permitted Sterne spores to germinate and grow in broth used for viability testing. +/− indicates presence and/or absence of growth in broth/on plates. PBS, phosphate-buffered saline. †50% of tissue tested.

An alternative way to neutralize formaldehyde in fixed tissue is sufficient dilution in the broth used for viability testing. Thus, we determined the broth volume to which formaldehyde could be adequately diluted to permit growth of *B. anthracis* Sterne, *B. pseudomallei* 82, *F. tularensis* live vaccine strain, and *Y. pestis* Pgm–/pPst–. The growth of these non–Select Agents, used as surrogates, in broth containing 1%–0.001% formaldehyde, is shown in Table 7. Of note is the higher inoculum for *F. tularensis* live vaccine strain necessary to seed the broth cultures for growth. In contrast, substantially less inoculum was required for the other agents. *B. pseudomallei* 82 (a purine auxotroph) required longer incubation (5 d) before a turbidity increase was evident. Therefore, we also performed the formaldehyde sensitivity assay with the virulent strain 1026b. These data indicate that formaldehyde can be washed out or adequately diluted in broth to permit a small number of live organisms, which may be present, to proliferate.

**Table 7:** Growth of *Bacillus, Burkholderia, Francisella,* and *Yersinia* species in diluted formaldehyde in study of inactivation of bacterial Tier I Select Agents*

Strain	CFU/volume	Growth condition	Time, d	NBF concentration, %	Growth
*Bacillus anthracis* Sterne	5 (bacilli)/500 mL	BHI, 37°C	1	0.1	–
				0.01	+
				0.001	+
*Burkholderia pseudomallei* 82	10/500 mL	LB + 4% glycerol, 37°C	5	0.1	–
				0.01	–
				0.001	+
*B. pseudomallei* 1026b	5/500 mL	LB + 4% glycerol, 37°C	3	0.1	–
				0.01	+
				0.001	+
*Francisella tularensis* LVS	1.4 × 10^5^/100 mL	BHI + 1% IsoVitaleX, 37°C	4	0.1	–
				0.01	–
				0.001	+
*Yersina pestis* Pgm*^–^*/pPst*^–^*	10/100 mL	HI + 0.2% xylose, 28°C	3	0.1	–
				0.01	–
				0.001	+

*5 CFU *B. anthracis* Sterne (bacilli), 10 CFU *B. pseudomallei* 82, 5 CFU *B. pseudomallei* 1026b, 1.4 × 10^5^ CFU *F. tularensis* live vaccine strain and 10 CFU *Y. pestis* Pgm–/pPst– bacteria were inoculated into their respective broths containing 0.1%, 0.01%, and 0.001% NBF and grown at the indicated temperature. Time listed is when broth cultures reached turbidity. + /– indicates presence and/or absence of growth in broth. BHI, brain heart infusion; HI, heart infusion; LB, Lennox broth; LVS, live vaccine strain; NBF, neutral buffered formalin.

The skin is a difficult tissue for formaldehyde to infiltrate (*16*). Because of this property, along with the resistant nature of spores, we chose skin tissues infected with *B. anthracis* Ames spores to generate a time course of organism killing. Spores in skin sections fixed for 14 or 21 days were completely inactivated (Table 1). In contrast, 7-day fixation was not adequate; growth occurred in 1 of 3 tissues. To maximize our dataset for inactivation of spore-containing tissues, we also fixed lung tissues from rabbits infected with *B. anthracis* Ames spores. Of 3 lung samples, 2 were inactivated at 13 days and the third was inactivated at 20 days (Table 8).

**Table 8:** Formaldehyde fixation of infected lung tissue from rabbits exposed to aerosolized *Bacillus anthracis* Ames spores in study of inactivation of bacterial Tier I Select Agents*

Rabbit no.	Tissue weight, g	Tissue size, mm	Total CFU recovered/g	Heat-resistant CFU recovered/g	Time of inactivation		Positive control
					13 d	20 d	
1	0.72	10 × 10 × 10	1.2 × 10^7^	1.5 × 10^6^	–/–†	ND	+/+
2	0.74	10 × 10 × 10	4.4 × 10^5^	4.3 × 10^5^	–/–†	ND	+/+
3	0.71/0.75	10 × 10 × 10	2.6 × 10^6^	1.0 × 10^6^	+/+†	–/–†	+/+

*Infected lung tissues from 3 rabbits were fixed for 13 or 20 d in 4% paraformaldehyde, rinsed for 30 min in phosphate-buffered saline, and then homogenized. Listed are the tissue weight, size, and CFU recovered. +/– indicates presence and/or absence of growth in broth/on plate. ND, not done. †50% of sample tested.

The tissues that we commonly collect and fix for histopathologic analysis are spleen, liver, and lung. Organisms in all infected spleen, liver, and lung samples fixed for various times were inactivated (Table 2). Specifically, we found complete inactivation in spleens infected with the highest load of *B. anthracis* Ames, *F. tularensis* Schu S4, or *Y. pestis* CO92, tested by using either 90% or 100% of the samples. This inactivation includes *B. anthracis* Ames–infected lung tissue from guinea pigs, and rabbit, which also contained heat-resistant spores. Also completely inactivated were *B. pseudomallei* 1026b–infected spleen and other tissues, tested by using 50% of the samples. Similar to formaldehyde-fixed tissues, tissues incubated in EM fixative were also inactivated (Table 3).

## Discussion

We examined the effectiveness of formaldehyde, by itself and with glutaraldehyde, to inactivate tissues infected with Select Agents. We found that 14 days in formaldehyde and 7 days in EM fixative are generally sufficient to completely inactivate most infected tissues described in this report, including tissues containing high numbers of resistant spores and hard-to-infiltrate tissues like skin. One exception was a rabbit lung in which *B. anthracis* spores were only partially inactivated at 13 days of fixation but inactivated at 20 days. Although we show that inactivation can probably be achieved in less time, to ensure an adequate safety margin, no change in our institutional standard operating procedure of 21 days fixation will be made.

At the study’s inception, we experimented with injecting Select Agents into tissues collected from euthanized animals. However, this ex vivo approach failed because the tissues did not retain the inoculum. Of note, this attempt did not recapitulate in vivo diseased tissue, where organisms are probably distributed more homogeneously. Although we were fortunate to obtain rabbit lung tissues from an ongoing study, other already infected tissues were not readily available. Thus, we infected animals specifically for this work. In addition to infecting with the Select Agents we commonly work with, we also validated different tissue types such as skin and lung, tissues in which a substantial amount of ungerminated spores would remain after exposure. We specifically examined the number of ungerminated spores in rabbit and guinea pig tissues because the chemical sensitivities of spores and bacilli differ greatly.

For this study, we used tissues <10 mm thick in 1 dimension. Although it is possible to excise and fix tissue >10 mm thick, this practice is discouraged because it hampers fixative infiltration into the deep recesses of the tissue; these areas probably also undergo putrefaction before becoming fixed (*10*). Infiltration is already slowed in tissues such as skin and fat (*16*,*17*), so exceeding the limit set forth here (10 mm) will probably lengthen the time needed to inactivate. Exceeding the organ bacterial burdens greater than those stated (Tables 2,3) would also require reevaluation (*23*) because these could require more time to inactivate. Other institutions may use this work as a guide to conduct and develop their own testing to comply with the policy. Furthermore, these methods may be useful in the processing and inactivation of tissues from patients infected with Select Agents for diagnostic testing by state public health laboratories and CDC.
